# 

**Published:** 1869-05

**Authors:** 


### CONTENTS.

ORIGINAL COMMUNICATIONS.

Address........ 185
Dental Squibs........ 188
Manipulating Foil........ 190
To Prevent Vulcanizers from Leaking Steam........ 192
Chloro-nitrous Oxide........ 194
Rimming Plates........ 194

PROCEEDINGS OF SOCIETIES.

Report of discussions at the twenty-fourth annual meeting of the Mississippi Valley Dental Association, March 4, 5 and 6, 1868........ 197
Wabash Valley Dental Society........ 212
Harris Dental Association........ 213
Constitution of the American Dental Association........ 214
Dental Association........ 223

SELECTIONS.

Sulphate of Nickel.—A Sedative........ 224
Third Dentition........ 224
A Case of Petrifaction........ 225
Two Cases of Death from Chloroform........ 225
A Superior Liquid Glue........ 225
Treatment of Diseased Gums........ 226
The Last Wonder of the Spectroscope........ 226
Leeches........ 226

EDITORIAL.

Sore, or Sour?........ 227

